# Identification of co-segregating *GJA3* and *CRYBA1* missense variants in a Chinese family with congenital cataract: a possible digenic etiology

**DOI:** 10.3389/fmed.2025.1743231

**Published:** 2026-01-13

**Authors:** Chenchen Zhou, Kunke Li, Zhenxing Zhou, Shuhui Jian, Ling Jin, Chenghu Wang, Xiaoqian Zhang

**Affiliations:** 1 The Affiliated Eye Hospital, Nanjing Medical University, Nanjing, China; 2 Shenzhen Eye Hospital, Shenzhen Eye Medical Center, Southern Medical University, Shenzhen, China

**Keywords:** congenital cataract, *CRYBA1*, *GJA3*, missense mutation, whole-exome sequencing

## Abstract

**Introduction:**

Congenital cataract (CC), defined as lens opacity present at birth or in early infancy, is a major cause of reversible childhood blindness and shows marked genetic heterogeneity. This study aimed to investigate the genetic basis of CC in a multigenerational Chinese family.

**Methods:**

A four-generation family with CC was clinically characterized. Whole-exome sequencing was performed in the proband, followed by stepwise variant filtering based on minor allele frequency, predicted functional impact, known CC-associated genes, and an autosomal dominant inheritance model. Candidate variants were annotated and classified according to ACMG guidelines. Sanger sequencing was used to validate variants in two additional affected relatives.

**Results:**

Two heterozygous missense variants were identified in known CC-associated genes: *GJA3* c.776C > A (p.Ser259Tyr) and *CRYBA1* c.346A > T (p.Ile116Phe). Both were extremely rare or absent in population databases and predicted to be damaging by multiple in silico tools. Sanger sequencing confirmed that the two variants co-occurred in all three affected family members tested, and no other rare, protein-altering variants meeting the filtering criteria were found in established cataract genes.

**Discussion:**

According to ACMG guidelines, both variants remain classified as variants of uncertain significance, but their rarity, predicted functional impact and consistent co-occurrence in affected individuals support them as strong candidate variants that may jointly contribute to CC in this family and expand the spectrum of GJA3- and CRYBA1-associated changes.

## 1 Introduction

Congenital cataract (CC), defined as lens opacity present at birth or during early infancy, is a leading cause of childhood blindness and amblyopia. The global prevalence of CC is estimated at approximately 1–6 per 10,000 live births, representing a significant contributor to visual impairment, particularly in developing countries (1, 2). Etiologically, around one-third of CC cases are caused by genetic factors, whereas the remaining cases may result from intrauterine infections (e.g., rubella virus or cytomegalovirus), metabolic disorders (e.g., galactosemia), drug exposure, or other environmental insults (3, 4).

Hereditary congenital cataract exhibits considerable genetic and phenotypic heterogeneity. To date, over 100 causative genes reported to be associated with CC have been identified, with autosomal dominant inheritance being the most common mode of transmission. The proteins encoded by these genes perform diverse biological functions, which can be broadly grouped into several major categories: (1) structural crystallins (e.g., *CRYAA*, *CRYBB1*, *CRYBA1*), which are essential for maintaining lens structural stability and optical transparency (5–7); (2) gap junction proteins (e.g., *GJA3*, *GJA8*), which mediate the intercellular exchange of ions and small metabolites among lens fiber cells (8–11); (3) membrane transport proteins (e.g., *MIP*, *SLC16A12*), which regulate transmembrane transport of water and small molecules to maintain osmotic homeostasis within lens cells (12, 13); and (4) transcription factors (e.g., *PAX6*, *MAF*), which precisely control the expression of key structural proteins during lens development and differentiation (14–16). Mutations in any of these functional categories can disrupt lens structural integrity or metabolic balance, ultimately leading to loss of transparency and cataract formation (17, 18).

Among the numerous genes implicated in congenital cataracts, *GJA3* and *CRYBA1* represent two prototypical and functionally distinct categories of genes that have been repeatedly linked to CC (8). The *GJA3* gene encodes connexin 46 (Cx46), a major gap junction protein expressed in lens fiber cells. Cx46 forms intercellular channels that facilitate the exchange of ions and small metabolites, which is essential for maintaining lens metabolic homeostasis and transparency. Pathogenic mutations in *GJA3* can impair channel function or alter protein conformation, leading to ionic imbalance and abnormal protein aggregation within lens cells—an important mechanism underlying autosomal dominant congenital cataracts (19, 20). The *CRYBA1* gene encodes βA1/A3-crystallin, a major water-soluble structural protein crucial for maintaining lens integrity and refractive properties. Mutations in *CRYBA1* often result in protein misfolding, abnormal aggregation, or reduced stability, thereby disrupting lens optical homogeneity and ultimately causing lens opacification (21, 22).

Previous studies have reported multiple pathogenic mutations in *GJA3* and *CRYBA1* across diverse populations (20, 22, 23). However, families carrying concurrent mutations in both genes are exceedingly rare, and their associated clinical and genetic features remain poorly characterized. Therefore, the use of a combined approach involving linkage analysis and next-generation sequencing (NGS) to identify novel or co-occurring variants in rare congenital cataract pedigrees is of considerable significance. Such efforts can enhance our understanding of the molecular mechanisms underlying congenital cataracts, expand the known mutation spectrum, and improve the precision of genetic counseling.

In this study, we performed a comprehensive genetic analysis of a multigenerational Chinese family affected by congenital cataract. High-throughput whole-exome sequencing (WES) was employed to screen for potential candidate variants, followed by co-segregation analysis within the family to confirm their genetic relevance. This study aimed to characterize the genetic variants identified in this pedigree and to explore their potential contribution to the observed cataract phenotype.

## 2 Materials and methods

### 2.1 Patient recruitment and DNA extraction

A multigenerational Chinese family affected by congenital cataract was recruited for this study. Three affected members underwent detailed ophthalmic examinations at the Eye Hospital of Nanjing Medical University, while a total of 27 family members received comprehensive clinical evaluations, including medical history review, visual acuity assessment, intraocular pressure measurement, and slit-lamp biomicroscopy. Thirteen individuals were diagnosed with congenital cataract, and pedigree analysis suggested an autosomal dominant inheritance pattern. All participants provided written informed consent for the use and publication of their clinical and genetic data. The study was conducted in accordance with the principles of the Declaration of Helsinki and was approved by the Ethics Committee of the Eye Hospital of Nanjing Medical University. Peripheral blood samples were collected from the three clinically affected members (II:2, III:2, and IV:1), and genomic DNA was extracted using the DNeasy Blood & Tissue Kit (Qiagen, Cat. No. 69506). Whole-exome sequencing (WES) was performed only in the proband (III:2), while the two additional affected relatives (II:2 and IV:1) underwent Sanger sequencing for validation of candidate variants. DNA purity and concentration were evaluated before library preparation.

### 2.2 Exome sequencing and bioinformatic analysis

Genomic DNA was extracted from the proband’s peripheral blood using the DNeasy Blood & Tissue Kit (Qiagen, Cat. No. 69506). An exome library was prepared using IDT xGen^®^ Exome Research Panel v1.0 and the IDT xGen^®^ Human mtDNA Research Panel and sequenced on the Illumina NovaSeq 6000 platform (PE150). The target regions achieved a coverage of 99.92%, with an average sequencing depth of 151×, and the mitochondrial genome reached an average depth of 11,899×, enabling reliable detection of single-nucleotide variants (SNVs) and small insertions/deletions (INDELs) within the exome. Burrows–Wheeler Aligner (BWA) was used to align sequencing reads to the human reference genome (GRCh37), and variant calling was performed using the Genome Analysis Toolkit (GATK). Variants were annotated using ANNOVAR, followed by stepwise filtering: (1) removal of low-quality variants; (2) exclusion of variants with minor allele frequency (MAF) > 1% in population databases (dbSNP, 1000 Genomes East Asian subset, ExAC_EAS, gnomAD_exome_EAS); (3) retention of exonic and canonical splice-site variants (±2 bp); (4) prioritization of protein-altering variants (missense, nonsense, frameshift, or splice-site); and (5) selection of heterozygous variants consistent with the assumed autosomal dominant inheritance model. Gene–phenotype correlations were further assessed using ClinVar^1^ and HGMD^2^ databases. Variant classification and interpretation were performed according to the American College of Medical Genetics and Genomics (ACMG) guidelines. *In silico* pathogenicity predictions, including assessments of evolutionary conservation and functional impact, were performed using REVEL, SIFT, MutationTaster, PolyPhen-2, PROVEAN, ClinPred, and LRT. Two heterozygous missense variants identified in the proband through WES were subsequently validated in two additional affected relatives (II:2 and IV:1) by Sanger sequencing.

### 2.3 PCR amplification and sanger sequencing

The reference sequences of exon 2 of the *GJA3* gene and exon 4 of the *CRYBA1* gene were retrieved from the NCBI GenBank database. Specific primer pairs were designed to amplify the regions containing the candidate variants c.776C > A (p.Ser259Tyr) in *GJA3* and c.346A > T (p.Ile116Phe) in *CRYBA1*. The primer sequences for *GJA3*, forward primer 5′-GTTAGG AATCTGAAGCAATGGGC-3′ and reverse primer 5′-GGGAT CGGCTGTCCCCAG-3′; for *CRYBA1*, forward primer 5′-CTGG ATTGGTTATGAGCATACCAG-3′ and reverse primer 5′-AGCT GAACAGATGGGGCGG-3′. PCR amplification was performed using these primers under standard conditions recommended by the manufacturer of the DNA polymerase and the resulting products were subjected to Sanger sequencing to validate the presence of the two candidate missense variants in the three affected family members (II:2, III:2, and IV:1).

### 2.4 Statistical analysis

All statistical analyses were conducted using R software (version 4.4.1) and the SangerBox 2 online platform. Genotype–phenotype co-segregation was assessed descriptively based on the consistent co-occurrence of the two missense variants among the three affected family members.

## 3 Result

### 3.1 Clinical manifestations of the family

**FIGURE 1:**
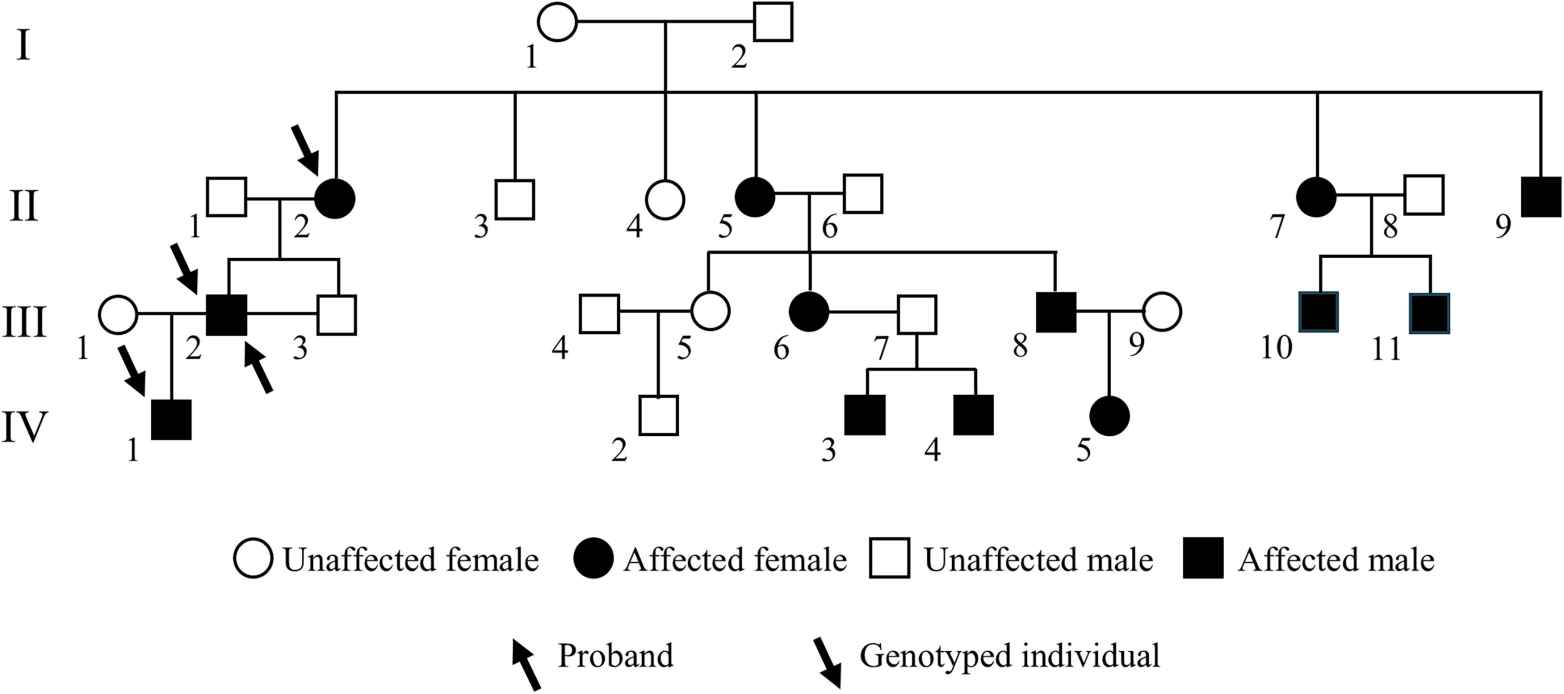
Pedigree of a four-generation Chinese family with congenital cataracts. The arrow indicates the proband. Squares represent males and circles represent females; filled symbols denote affected individuals, and open symbols denote unaffected individuals.

**FIGURE 2:**
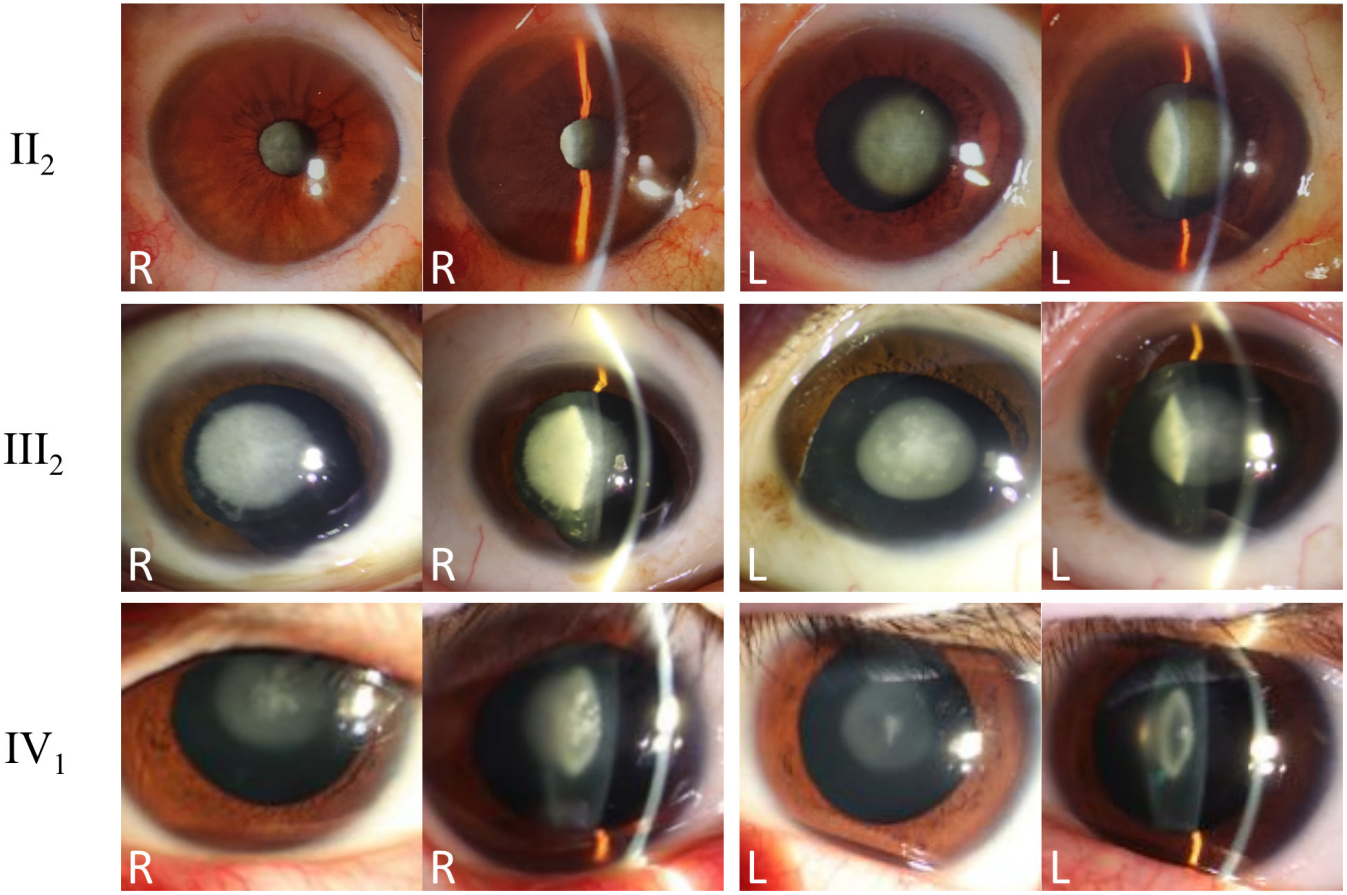
Clinical phenotypes and slit-lamp examination findings of affected family members. Slit-lamp anterior segment photographs of the proband (III:2), his mother (II:2), and his son (IV:1) are presented. Both direct and oblique illumination views of both eyes are shown, revealing typical nuclear opacities consistent with congenital cataracts in all affected individuals.

This study included a four-generation Chinese family affected by congenital cataracts. Pedigree analysis indicated that affected individuals were present from the second generation onward, with a similar proportion of affected males and females (Figure 1), consistent with an autosomal dominant inheritance pattern and preliminarily excluding X-linked inheritance. The proband (III:2), a 53-year-old male, exhibited marked lens opacity in the right eye and mild lens opacity in the left eye, characteristic of congenital cataract. His mother (II:2, 76 years old) also presented with varying degrees of bilateral lens opacification. The proband’s son (IV:1, 22 years old) showed bilateral nuclear cataracts, with the right eye being more severely affected (Figure 2).

A detailed summary of clinical features—including cataract morphology, severity, visual acuity, and systemic conditions—was compiled for all 13 affected members (Table 1). Across the family, nuclear or perinuclear lens opacities were the most common morphological pattern, although some individuals displayed mixed or cortical involvement. The severity of lens opacity generally increased with age, and younger affected individuals often showed milder lens changes compared with older family members, indicating a clear age-dependent progression of the cataract phenotype.

**TABLE 1:** Clinical characteristics of the 13 affected members in the Chinese congenital cataract family.

Individual ID	Age	Sex	Cataract morphology	Surgical history (R/L)	Systemic disease
II:2	76	Female	Severe nuclear	Yes/yes	No
II:5	78	Female	Total	Yes/yes	Diabetes
II:7	73	Female	Severe nuclear + perinuclear	Yes/yes	Hypertension
II:9	75	Male	Severe nuclear	Yes/yes	No
III:2 (proband)	53	Male	Moderate nuclear + perinuclear	Yes/yes	No
III:6	56	Female	Severe nuclear	Yes/yes	Hypertension
III:8	58	Male	Moderate nuclear + perinuclear	Yes/no	No
III:10	53	Male	Moderate nuclear	No/yes	Hyperlipidemia
III:11	50	Male	Severe nuclear + Perinuclear	Yes/yes	Diabetes
IV:1	22	Male	Moderate nuclear + Perinuclear	Yes/yes	No
IV:3	30	Male	Moderate nuclear	No/yes	No
IV:4	34	Male	Moderate nuclear	Yes/no	No
IV:5	32	Female	Mild nuclear + perinuclear	No/no	No

Nuclear, nuclear cataract; perinuclear, perinuclear opacity; total, total cataract. “Surgical history (R/L)” indicates whether cataract extraction surgery was performed in the right (R) and/or left (L) eye.

### 3.2 Inheritance pattern identification and co-occurrence analysis

**FIGURE 3:**
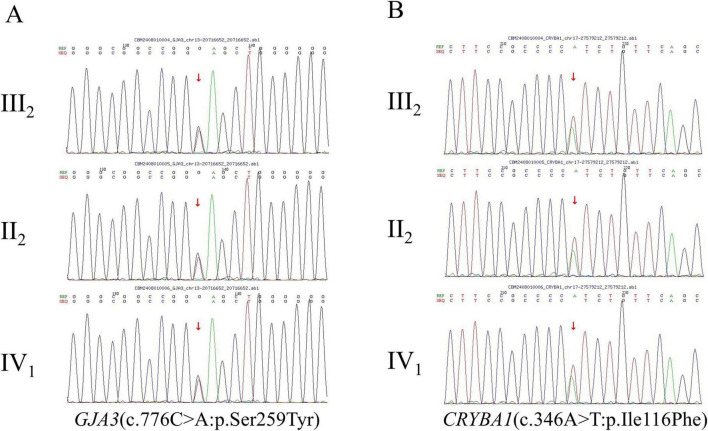
Sanger sequencing validation of *GJA3* and *CRYBA1* variants in the family. **(A)** DNA sequencing chromatograms of family members (II:2), (III:2), and (IV:1) show a heterozygous missense variant c.776C > A (red arrow) in exon 2 of GJA3, resulting in an amino acid substitution of serine to tyrosine at position 259 (transcript: NM_021954.4). **(B)** DNA sequencing chromatograms of family members (II:2), (III:2), and (IV:1) reveal a heterozygous missense variant c.346A > T (red arrow) in exon 4 of CRYBA1, leading to an amino acid substitution of isoleucine to phenylalanine at position 116 (transcript: NM_005208.5).

High-throughput whole-exome sequencing of the proband identified heterozygous missense variants in two known cataract-associated genes. In *GJA3*, the causative gene for autosomal dominant congenital cataract type 14 (Cataract 14, AD), a heterozygous missense variant c.776C > A (p.Ser259Tyr) was detected in exon 2 (transcript: NM_021954.4), one of the two exons comprising the gene. In *CRYBA1*, the causative gene for autosomal dominant congenital cataract type 10 (Cataract 10, AD), a heterozygous missense variant c.346A > T (p.Ile116Phe) was identified in exon 4 (transcript: NM_005208.5), one of the six exons of the gene. Sanger sequencing of all available family members confirmed that both *GJA3* (c.776C > A) and *CRYBA1* (c.346A > T) variants were heterozygous in the proband (III:2), his affected mother (II:2), and his affected son (IV:1), and that these variants showed consistent co-occurrence among the three affected individuals, consistent with an autosomal dominant inheritance pattern (Figure 3).

### 3.3 Bioinformatic analysis and pathogenicity assessment of the variants

Bioinformatic analyses provided important supportive evidence for evaluating the two candidate variants. The *GJA3* c.776C > A (p.Ser259Tyr) variant was predicted to be deleterious by SIFT, whereas other in-silico tools, including REVEL (0.342) and MutationTaster (“polymorphism”), did not provide consistent pathogenic support; importantly, this variant was absent from all major population databases. The *CRYBA1* c.346A > T (p.Ile116Phe) variant was supported by multiple computational predictions, including SIFT (“damaging”), MutationTaster (“disease causing”), and a moderate REVEL score of 0.587, and showed an extremely low allele frequency in the East Asian subset of the gnomAD database (MAF = 0.0003) (Table 2).

**TABLE 2:** Characteristics of the identified *GJA3* and *CRYBA1* variants.

Gene	Locus	Associated diseases	MAF	Proband genotype	ACMG evidence	Protein structure prediction
*GJA3*	NM_021954.4 EXON(2/2) c.776C > A p.Ser259Tyr	Cataract 14 (AD)	0	Heterozygote	PM2	Revel: 0.342, SIFT: damaging, splicing prediction: MutationTaster_pred: polymorphism.
*CRYBA1*	NM_005208.5 EXON(4/6) c.346A > T p.Ile116Phe	Cataract 10 (AD)	0.0003	Heterozygote	PM2 + PP3	Revel: 0.587, SIFT: damaging, splicing prediction: MutationTaster_pred: Disease_causing.

MAF values are derived from the gnomAD v2.1.1 East Asian population (“0” indicates absence in the database). ACMG evidence was evaluated according to the American College of Medical Genetics and Genomics guidelines. Protein structure predictions summarize *in silico* assessments from REVEL, SIFT, and MutationTaster. AD, autosomal dominant; MAF, minor allele frequency.

According to the ACMG guidelines, both variants remain classified as variants of uncertain significance (VUS)—with *GJA3* c.776C > A (p.Ser259Tyr) supported by PM2 and *CRYBA1* c.346A > T (p.Ile116Phe) supported by PM2 and PP3. Nonetheless, their consistent co-occurrence among the three affected family members who underwent genetic testing, together with their functional relevance to lens biology, supports their candidacy as potential contributors to the cataract phenotype in this pedigree.

### 3.4 Additional genetic analysis

As a complement to whole-exome sequencing, mitochondrial genome sequencing was performed to investigate variants in mitochondrial DNA. No clearly pathogenic variants were detected in the mitochondrial genome. This negative finding helps exclude mitochondrial etiologies and supports considering the *GJA3* and *CRYBA1* variants as the most plausible candidate variants in this pedigree.

## 4 Discussion

Congenital cataract is one of the leading causes of visual impairment and treatable blindness in children. Its clinical manifestations are highly heterogeneous, with common types including nuclear and perinuclear cataracts, lamellar cataracts, and congenital diffuse cataracts, each of which can affect visual function to varying degrees (3, 24). The disease exhibits marked genetic heterogeneity, and over 100 disease-associated genes have been identified to date, involving multiple key biological processes such as lens development, structural maintenance, and metabolic homeostasis (10, 25, 26). Despite the identification of numerous pathogenic genes, approximately 30%–50% of cases remain without a definitive molecular diagnosis based on known genes, suggesting the existence of yet-to-be-discovered pathogenic genes or more complex genetic mechanisms.

*GJA3* is located on chromosome 13 and encodes the gap junction protein α3 (connexin 46), which plays a critical role in maintaining normal communication among lens fiber cells and forming functional gap junction channels (3, 27). These channels are essential for regulating lens osmotic pressure and metabolic homeostasis, as well as for the transport of nutrients and metabolic waste, thereby contributing to lens transparency (28). Mutations in *GJA3* may alter the conformation of connexin proteins, disrupt the formation of lens fibers, and ultimately lead to congenital cataracts. Previous studies have reported that *GJA3* mutations are associated with autosomal dominant lamellar and zonular pulverulent cataracts. For example, Boese et al. described a four-generation family with congenital cataracts carrying a *GJA3* variant (NM_021954.4: c.199G > T, p.Asp67Tyr), which may increase the risk of glaucoma and retinal detachment following cataract surgery (8). Additionally, Zhu et al. identified a c.671A > G mutation in *GJA3*, which altered channel protein activity and led to congenital perinuclear cataracts (23). In the present study, we identified a *GJA3* variant (c.776C > A, p.Ser259Tyr) that is observed to co-occur with the cataract phenotype in affected family members with congenital cataract type 14, and the affected individuals exhibited typical clinical features, including bilateral cataracts, visual impairment, and nuclear lens opacities (21). Nuclear lens opacity is one of the most common forms of congenital cataracts, usually localized to the central region of the lens (embryonic and fetal nuclei) and appearing as a disc-like opacity surrounding the transparent nuclear zone (29, 30). Because this location is near the optical center, it significantly affects vision, making this type of cataract more easily detected and diagnosed.

*CRYBA1* is located on chromosome 17 and encodes a highly conserved βA1 soluble crystallin, which exists in an oligomeric state within the lens (31). Mutations in *CRYBA1* may cause structural abnormalities, disrupt normal protein folding and assembly, and thereby impair lens development and transparency. To date, 308 pathogenic variants affecting crystallin proteins have been identified, accounting for nearly 23% of all hereditary cataract mutations (32). Early studies demonstrated that deletions in exon 4 of *CRYBA1* (c.271–273delGAG and c.269–271del, p.G91del) result in the loss of a highly conserved glycine residue, leading to nuclear lamellar cataracts, significantly reducing gene expression and promoting protein aggregation at the plasma membrane in affected individuals (33, 34). In the present study, we identified a heterozygous missense variant in *CRYBA1* (c.346A > T, p.Ile116Phe), which, although classified as a variant of uncertain significance (VUS) according to ACMG guidelines, exhibits extremely low population frequency (PM2), follows the observed familial inheritance pattern, and is predicted to be deleterious by multiple bioinformatic tools (PP3), including a REVEL score of 0.587, SIFT prediction as deleterious, and splice site predictions indicating pathogenicity. These findings support c.346A > T (p.Ile116Phe) variant as a plausible candidate variant that may contribute to the cataract phenotype in this family and may help expand the spectrum of *CRYBA1*-associated variants.

In classical monogenic inheritance, disease is typically driven by mutations in a single gene. However, the most striking feature of the family studied here is the simultaneous presence and complete co-segregation of variants in *GJA3* and *CRYBA1*, suggesting a possible digenic interaction rather than confirming a strict digenic inheritance model. Digenic inheritance refers to a mechanism in which the manifestation of a phenotype requires the combined effect of mutations in two different genes, and this mechanism has been documented in various hereditary eye diseases (35, 36). For example, Liu et al. reported that heterozygous loss-of-function mutations in *RP1L1* and C2orf71 act through genetic interaction to jointly cause syndromic retinal degeneration (37). Kelberman et al. described a digenic pattern involving *PITX2* and *FOXC1*, where mutations in both genes resulted in a more severe Axenfeld-Rieger syndrome phenotype (38). Blickhäuser et al. proposed that the mitochondrial variant m.11778G > A acts in combination with heterozygous pathogenic variants in nuclear genes encoding subunits of mitochondrial complex I, explaining a digenic mechanism underlying Leber hereditary optic neuropathy (LHON) in the context of the Leigh syndrome spectrum (LSS) (39).

In the present study, the *GJA3* mutation primarily affects intercellular communication and metabolic homeostasis in lens cells, whereas the *CRYBA1* mutation mainly disrupts the stability and solubility of structural proteins. These two mutations therefore impact lens homeostasis through distinct functional and structural pathways. This combination of effects may contribute jointly to the cataract phenotype observed in this family. Such a potential dual-gene effect provides a plausible explanation for the clinical presentation but does not constitute functional proof of digenic inheritance. Overall, the findings point to a possible digenic contribution rather than a definitively established digenic mechanism, and highlight the importance of considering gene–gene interactions in the molecular diagnosis of congenital cataracts.

In summary, this study is among the first to report a co-occurrence pattern involving heterozygous variants in *GJA3* (c.776C>A) and *CRYBA1* (c.346A>T) in a Chinese family with congenital cataracts. Family analysis demonstrated that these two variants were observed together in all affected individuals tested, and both bioinformatic predictions and population frequency data support their potential pathogenic relevance. We propose a possible digenic contribution in which the GJA3 variant disrupts intercellular communication in lens cells, while the *CRYBA1* variant affects the stability of structural proteins, with their combined effects potentially contributing to cataract formation. This finding expands the mutation spectrum of *GJA3* and *CRYBA1*, provides new insights into the genetic mechanisms underlying congenital cataracts, and offers a reference for genetic counseling and clinical diagnosis, highlighting the importance of considering potential gene-gene interactions in clinical practice.

Finally, several aspects of this study will benefit from further optimization and validation in future work. First, genetic testing was performed only in affected family members, and unaffected relatives were not genotyped; therefore, a more stringent segregation analysis could not be carried out. Second, according to ACMG guidelines, the GJA3 and CRYBA1 variants identified in this family are still classified as variants of uncertain significance (VUS), and the current evidence is not sufficient for formal reclassification. Third, although the co-occurrence of these variants in affected individuals and the *in silico* predictions support their candidacy for a pathogenic role, functional studies have not yet been performed to directly verify their effects or to confirm a digenic interaction, which would provide stronger mechanistic evidence in future research.

## Data Availability

The whole-exome sequencing data from this study have been formally archived and are available in the GSA-Human repository of the China National Center for Bioinformation (CNCB) under the following accession number: HRA015396. The dataset can be accessed at the following link: https://ngdc.cncb.ac.cn/gsa-human/browse/HRA015396.

## Footnotes

1
https://www.ncbi.nlm.nih.gov/clinvar

2
https://www.hgmd.cf.ac.uk/
